# Five-year follow-up mortality prognostic index for colorectal patients

**DOI:** 10.1007/s00384-023-04358-0

**Published:** 2023-03-09

**Authors:** Miren Orive, Irantzu Barrio, Santiago Lázaro, Nerea Gonzalez, Marisa Bare, Nerea Fernandez de Larrea, Maximino Redondo, Sarai Cortajarena, Amaia Bilbao, Urko Aguirre, Cristina Sarasqueta, José M. Quintana, for the REDISSEC-CARESS/CCR group

**Affiliations:** 1 Department of Social Psychology, Faculty of Pharmacy, University of the Basque Country UPV/EHU, Vitoria, Spain; 2 Department of Mathematics, University of the Basque Country UPV/EHU, Leioa, Spain; 3 Servicio de Cirugía General, Hospital Basurto, Bilbao, Bizkaia Spain; 4 Unidad de Investigación, Hospital Galdakao-Usansolo, Galdakao, Bizkaia Spain; 5 Unidad de Epidemiología Clínica, Corporacio Parc Tauli, Barcelona, Spain; 6 Centro Nacional de Epidemiología, ISCIII, Madrid, Spain; 7 Unidad de Investigación, Hospital Costa del Sol, Malaga, Spain; 8 Unidad de Investigación, Hospital Basurto, Bilbao, Bizkaia Spain; 9 Unidad de Investigación, Hospital Donostia/BioDonostia, Donostia, Gipuzkoa Spain; 10 Red de Investigación en Servicios Sanitarios y Enfermedades Crónicas (REDISSEC), Galdakao, Spain; 11 CIBER Epidemiología y Salud Pública (CIBERESP), Madrid, Spain; 12 Red de Investigación en Cronicidad, Atención Primaria y Promoción de La Salud (RICAPPS), Galdakao, Spain; 13 Basque Center for Applied Mathematics, BCAM, Bilbao, Spain; 14 Kronikgune Institute for Health Services Research, Barakaldo, Spain

**Keywords:** Colorectal cancer, Patient-reported outcome measures, Health-related quality of life, Cohort studies, Survival models

## Abstract

**Purpose:**

To identify 5-year survival prognostic variables in patients with colorectal cancer (CRC) and to propose a survival prognostic score that also takes into account changes over time in the patient’s health-related quality of life (HRQoL) status.

**Methods:**

Prospective observational cohort study of CRC patients. We collected data from their diagnosis, intervention, and at 1, 2, 3, and 5 years following the index intervention, also collecting HRQoL data using the EuroQol-5D-5L (EQ-5D-5L), European Organization for Research and Treatment of Cancer’s Quality of Life Questionnaire-Core 30 (EORTC-QLQ-C30), and Hospital Anxiety and Depression Scale (HADS) questionnaires. Multivariate Cox proportional models were used.

**Results:**

We found predictors of mortality over the 5-year follow-up to be being older; being male; having a higher TNM stage; having a higher lymph node ratio; having a result of CRC surgery classified as R1 or R2; invasion of neighboring organs; having a higher score on the Charlson comorbidity index; having an ASA IV; and having worse scores, worse quality of life, on the EORTC and EQ-5D questionnaires, as compared to those with higher scores in each of those questionnaires respectively.

**Conclusions:**

These results allow preventive and controlling measures to be established on long-term follow-up of these patients, based on a few easily measurable variables.

**Implications for cancer survivors:**

Patients with colorectal cancer should be monitored more closely depending on the severity of their disease and comorbidities as well as the perceived health-related quality of life, and preventive measures should be established to prevent adverse outcomes and therefore to ensure that better treatment is received.

**Trial registration:**

ClinicalTrials.gov identifier: NCT02488161.

**Supplementary Information:**

The online version contains supplementary material available at 10.1007/s00384-023-04358-0.

## Introduction

Colorectal cancer (CRC) continues to be a major health burden, with high mortality rates throughout the world [1]. It ranks as the second most common cancer cause of death worldwide, leading to more than 881,000 deaths in 2018 [1, 2]. It is therefore important to identify factors that help predict the prognosis in CRC patients [3]. As recently stated by Vogelsang et al. [4], individualized risk assessment provides opportunities for tailoring treatment strategies, optimizing healthcare resources, and improving outcomes [4]. Ideally, these would be factors that can be measured quickly, easily, and non-invasively in an ordinary clinical setting [3].

Several scoring systems have been developed to predict operative risk [5], such as the estimation of physiologic ability and surgical stress (E-PASS) score [6], the colorectal physiologic and operative severity score for the enumeration of mortality (CR-POSSUM) [7], and the prognostic nutritional index (PNI) [8]. Models also exist that target early stages in the postoperative period, such as 30-day and 90-day mortality predictions after CCR surgery [4]. However, these scoring systems and models evaluate patients’ physiological condition and outcomes in the short term, and none of them takes into account patient-reported outcomes.

Health-related quality of life (HRQoL) is emerging not only as an important outcome in survivorship care, but also as a factor that influences mortality [6, 9]. Better HRQoL has been found to be associated with a lower risk of dying [6, 10]. Many articles deal with this subject, but as Mosher et al. mention in a review [11], gaps remain, especially during long-term CCR. Apart from this, those prior studies primarily assessed HRQoL in patients with advanced stage of the disease [12–16], or they did not monitor tumor stage, recurrence, or patients’ comorbidities [17]. In our previous study [10], we observed that non-survivors had poorer anxiety, depression, and HRQoL scores than survivors during the 5-year follow-up, and the baseline scores were worse in those closest to death and worsened as death approached. Longitudinal assessment of patients is therefore important for management of such patients, as supported by the literature, specifically Sepehrvand et al. [18]. On the other hand, the overall 5-year relative survival rate for colorectal cancer is 65% [19].

For the above reasons, it can be concluded that there is still a need for a simple instrument that will enable evaluation of the risk of mortality for colorectal cancer in the years following surgery and that includes an update of the patients’ HRQoL over time in a survival analysis. The aims of this research were twofold: first, to identify significant mortality prognostic variables in patients with CRC among time-dependent patient-reported outcomes as well as basal clinical and sociodemographic variables; and second, to propose a survival prognostic score which considers changes over time in patient’s quality of life status. The use of the prognostic score will provide an estimate of the risk of mortality at a given time (within 5 years of the intervention) which could aid clinicians in the management of CRC patients, thus improving their outcomes.

## Material and methods

### Patients

This was a longitudinal prospective observational analytic cohort study that includes patients from nineteen public hospitals representing nine provinces in Spain, all of which operate under the Spanish National Health Service (SNHS), which is responsible for most of the country’s population, with a planned patient follow-up period of 5 years. Patients with colon or rectal cancer scheduled to undergo surgery between June 2010 and December 2012 were informed of the aims of the study and invited to participate. Only patients who provided written informed consent were allowed to enroll in the study. The study was approved by the Basque Ethics Committee (Approval Number: 11/23/2010 and PI2014084) and all study data were kept confidential. Patients were deemed eligible for this study if they were on the surgical waiting list of one of the participating hospitals and had a diagnosis of surgically resectable colon or rectal cancer. Exclusion criteria were in situ cancer, an unresectable tumor, terminal disease, inability to respond to questionnaires for any reason, and any severe mental or physical conditions that might prevent the patient from responding to questionnaires, as well as failure to consent to participate. Detailed information of the protocol has been published elsewhere [20].

### Data collection

Clinical data and patient-reported outcome measures (PROMs) were collected at basal time (before surgery), and at 1, 2, 3 and 5 years after surgery. Data collected at hospital admission included sociodemographic data, clinical data (including information about comorbidities based on the Charlson comorbidity index), [21, 22] preoperative data, outpatient anesthesia data related to the surgical intervention, American Society of Anesthesiologists (ASA) class [23] pathology data including TNM stage and infiltrated lymph nodes, and data related to the period of admission after surgery (including the presence of complications, need for reoperation, readmission, and death). Clinical data were gathered from medical records and databases by qualified reviewers. In order to ensure consistency among centers and reviewers, instruction manuals were prepared to guide the data collection process.

Patients completed Spanish versions of the following patient-reported outcome measures (PROMs) before surgery and at 1, 2, 3, and 5 years after surgery:

Symptoms of anxiety and depression: The Hospital Anxiety and Depression Scale (HADS) [24, 25] was used, a specific questionnaire to measure symptoms of anxiety and depression in individuals with a physical illness. This is a 14-item measure: 7 items for depressive symptoms and 7 items for anxiety. A subscale score of 0 to 7 indicates absence of anxiety or depression; 8 to 10 a possible case of anxiety or depression; and 11 or higher a probable case of anxiety or depression.

Health-related quality of life: The European Organisation for Research and Treatment of Cancer’s Quality of Life Questionnaire Core 30 (EORTC-QLQ-C30) [26, 27] is a specific health-related quality of life questionnaire widely used in cancer research. It consists of 30 items that assess five functioning domains, eight cancer symptom domains, financial difficulties, and global quality of life. Scores are transformed to a 0-to-100 scale, with a high score on the Functional scale and on Global Quality of Life scale indicating better functioning and HRQoL. In this study, we considered The Global Quality of Life scale. The EuroQol-5D-5L (EQ-5D-5L) [28, 29] is a generic HRQoL measure which consists of two parts: (a) the descriptive system comprises 5-level Likert-type dimensions (mobility, self-care, usual activities, pain/discomfort, and anxiety/depression) with 5 answer options defining different levels of severity; and (b) the visual analogue scale (VAS) records the respondent’s self-rated health on a vertical, 20-mm visual analogue scale, ranging from 0 (worst imaginable state of health) to 100 (best imaginable state of health). For this study, only the descriptive system has been taken into account.

### Statistical analysis

The primary outcome of this study was the time between surgery and death. Those patients for whom the event of interest (death) was not observed during the 5-year study period were censored. All variables were measured at baseline, while PROMs were measured at baseline and at four points in time (1, 2, 3, and 5 years) after the intervention, all being assessed at each measurement point as a continuous variable. For exploratory analysis, the Kaplan–Meier survival curves were estimated for baseline patient characteristics [30]. Univariate Cox proportional hazard regression models were adjusted and variables with a *p*-value < 0.2 were included in the multivariate Cox proportional model. PROMs were introduced as time-dependent predictor variables, whose value was updated throughout the different measurements. The final multivariate model considered was the one whose variables had a significance level of alpha = 0.05 and greater discriminative ability measured by the c-index [31, 32], and the proportional hazard assumption was verified by means of the Schoenfeld global residuals test [33]. Due to the existing correlation between variables, not all the variables significant individually were significant in the multivariate model. We developed a time-dependent risk score by assigning a weight to each predictor variable in relation to the estimated *β* parameters based on the multivariate cox regression model. We then added up the risk weights of all the patient’s predictor variables, with higher scores indicating a greater probability of event at time *t*. The score was categorized into four risk groups by maximizing the c-index for the categorized variable using the catpredi package in R [34].The predictive accuracy of the risk score was assessed using the c-index.

Both the model and the score were validated. For the former, a tenfold cross-validation with 100 replicates was used to validate the stability of the estimated parameters as well as the c-index obtained. For the latter, a bootstrap validation was performed with 100 repetitions.

Statistical significance was assumed when *p* < 0.05. All statistical analyses were performed using R v.4.0.3.

## Results

In this study, 2531 patients were recruited who were followed up at 1, 2, 3, and 5 years, collecting sociodemogaphic and clinical information, mortality, and health-related quality of life. A flowchart describing the cohort evolution during the 5 years of follow-up is including in Supplementary Fig. 1. A description of baseline clinical characteristics of the sample is shown in Table 1. Changes in health-related quality of life over the 5 years of follow-up, as measured using the HADS, EORTC-Global Quality of Life Scale, and EQ-5D questionnaires, are shown in Table 2.

**Table 1:** Descriptive sociodemographic and clinical characteristics of CRC patients

**Variables**	***N*** **(%)**	**Missing**
**State**		
Censored	1806 (71.36%)	
Event	725 (28.64%)	
**Charlson’s comorbidity index**		
1–2	1341 (52.98%)	
3–4	934 (36.90%)	
≥ 5	256 (10.11%)	
**Log odds of positive nodes ratio**		148 (5.85%)
[0–0.04)	1528 (60.37%)	
[0.04–0.15)	399 (15.76%)	
[0.15–0.4)	302 (11.93%)	
[0.4–1]	154 (6.08%)	
**Gender**		
Male	1603 (63.33%)	
Female	928 (36.67%)	
**Age**		4 (0.16%)
< 70	1271(50.22%)	
[70, 80)	915 (36.15%)	
≥ 80	341 (13.57%)	
**pTNM**^**a**^		14 (0.55%)
0, I, II	1447 (57.17%)	
III	828 (32.71%)	
IV	242 (9.56%)	
**Location**		
Rectum	713 (28.17%)	
Colon	1818 (71.83%)	
**R stage**^**b**^		92 (3.63%)
R_0_	2218 (87.63%)	
R_1_	141 (5.57%)	
R_2_	80 (3.16%)	
**ASA**^**c**^		75 (2.96%)
I, II, III	2358 (93.16%)	
IV	98 (3.87%)	
**Invasion of other organs**^**d**^		
0 o 1	2245 (88.70%)	
2 o 3	286 (11.30%)	
**Treatment**		
None	2078 (82.10%)	
Chemo or radio	129 (5.10%)	
Chemo and radio	324 (12.80%)	

*N* frequency, *%* percentage

^a^pTNM: stage as classified by the American Joint Committee on Cancer 7th edition TMN system: I, the cancer has grown through the mucosa and has invaded the muscular layer of the colon. No regional lymph node metastasis or distant metastasis exists; II, the cancer has grown through the wall of the colon, or through the layers of the muscle to the visceral peritoneum, or has grown into nearby structures. No regional lymph node metastasis or distant metastasis exists; III, metastasis in regional lymph nodes but no distant metastasis. Cases with metastasis in regional lymph nodes and distant metastasis (IV) were excluded from further analysis

^b^R-stage of the operation: residual tumor (R) classification: R0, complete absence of tumor; R1, microscopic evidence of residual tumor; R2, macroscopic evidence of the tumor/palliative care; these cases were excluded from further analysis

^c^ASA: anesthesia risk in relation to patient’s condition, class I–III, from healthy patients to those with a serious systemic but not disabling disease; and IV, patients with a disabling serious systemic disease

^d^Invasion of other organs: number of types of invasion ( vascular, perineural, or lymphatic invasion)

**Table 2:** Descriptive statistics (absolute and relative frequencies) of the health-related quality of life scores in the four measurements over 5 years of follow-up

**Variables**	***N* (%)**				
	Basal	1st year	2nd year	3rd year	5th year
**HADS-Anxiety**					
≤ 7	1259 (49.74)	1207 (51.47)	1056 (48.51)	958 (47.10)	828 (45.85)
8–14	711 (28.09)	377 (16.08)	308 (14.15)	269 (13.23)	245 (13.57)
≥ 15	153 (6.05)	45 (1.92)	37 (1.70)	31 (1.52)	27 (1.50)
Missing	408 (16.12)	716 (30.53)	776 (35.65)	776 (38.15)	706 (39.09)
**HADS-Depression**					
≤ 7	1631 (64.44)	1345 (57.36)	1169 (53.70)	1016 (49.95)	910 (50.39)
8–14	415 (16.40)	252 (10.75)	202 (9.28)	213 (10.47)	185 (10.24)
≥ 15	75 (2.96)	41 (1.75)	35 (1.61)	29 (1.43)	27 (1.50)
Missing	410 (16.20)	707 (30.15)	771 (35.42)	776 (38.15)	684 (37.87)
**EuroQol-5D-5L**					
[− 0.1 to 0.6]	419 (16.55)	221 (9.42)	175 (8.04)	176 (8.65)	163 (9.03)
(0.6–0.8]	942 (37.22)	618 (26.35)	544 (24.99)	466 (22.91)	382 (21.15)
(0.8–1]	704 (27.82)	736 (31.39)	648 (29.77)	600 (29.50)	571 (31.62)
Missing	466 (18.41)	770 (32.84)	813 (37.21)	792 (38.94)	690 (38.21)
**EORTC-QLQ-C30**					
[0–54)	177 (6.99)	67 (2.86)	56 (2.57)	55 (2.70)	47 (2.60)
(54–78]	570 (22.52)	284 (12.11)	254 (11.67)	239 (11.75)	213 (11.79)
(78–88]	524 (20.70)	381 (16.25)	281 (12.91)	259 (12.73)	237 (12.12)
(88–100]	786 (31.05)	858 (36.59)	786 (36.10)	684 (33.63)	615 (34.05)
Missing	474 (18.73)	755 (32.20)	800 (36.75)	797 (39.18)	694 (38.43)
	*N* = 2531	*N* = 2345	*N* = 2177	*N* = 2034	*N = 1806*

*N* frequency, *%* percentage, *HADS-Anxiety* Hospital Anxiety and Depression questionnaire’s Anxiety subscale, *HADS-Depression* Hospital Anxiety and Depression questionnaire’s Depression subscale, *EORTC-QLQ-C30* The European Organisation for Research and Treatment of Cancer Quality of Life Questionnaire Core 30-Global Quality of Life scale

The results of the univariate analysis with the variables found to be predictors of mortality at different times of the 5-year follow-up of the cohort of patients with CRC are presented in Table 3. Based on these results, we selected the variables to be included in the multivariate model.

**Table 3:** Univariate Cox proportional hazards analysis of predictors of mortality over a 5-year follow-up

**Variables**	**Estimate**	**HR**	**HR(95% CI)**	***p***
**Charlson’s comorbidity index**				
1–2	Ref			
3–4	0.37	1.45	1.24 1.70	< 0.0001
≥ 5	0.96	2.61	2.12 3.21	< 0.0001
**Log odds of positive nodes ratio**				
[0–0.04)	Ref			
[0.04–0.15)	0.56	1.75	1.42 2.15	< 0.0001
[0.15–0.4)	1.00	2.71	2.21 3.33	< 0.0001
[0.4–1]	1.64	5.17	4.12 6.49	< 0.0001
Missing	1.07	2.93	2.24 3.83	< 0.0001
**Gender**				
M	0.21	1.23	1.06 1.44	0.008
F	Ref			
**Age**				
< 70	Ref			
[70, 80)	0.42	1.52	1.29 1.80	< 0.0001
≥ 80	1.12	3.07	2.54 3.71	< 0.0001
**pTNM**^**a**^				
0, I, II	Ref			
III	0.72	2.05	1.74 2.43	< 0.0001
IV	1.81	6.13	5.05 7.45	< 0.0001
**Location**				
Rectum	Ref			
Colon	0.04	1.04	0.88 1.23	0.626
**R stage**^**b**^				
R_0_	Ref			
R_1_	0.90	2.46	1.92 3.16	< 0.0001
R_2_	2.24	9.41	7.31 12.10	< 0.0001
Missing	0.72	2.06	1.49 2.85	< 0.0001
**ASA**^**c**^				
I, II, III	Ref			
IV	0.99	2.70	2.05 3.57	< 0.0001
Missing	0.81	2.24	1.61 3.11	< 0.0001
**Invasion of other organs**				
0 or 1	Ref			
2 or 3	0.98	2.67	2.23 3.19	< 0.0001
**Treatment**				
None	0.34	1.40	1.10 1.79	0.007
Chemo or radio	0.79	2.21	1.55 3.16	< 0.0001
Chemo and radio	Ref			
**HADS-Anxiety**				
≤ 7	Ref			
8–14	0.56	1.75	1.40 2.19	< 0.0001
≥ 15	0.65	1.92	1.23 2.99	0.004
Missing	1.22	3.38	2.84 4.02	< 0.0001
**HADS-Depression**				
≤ 7	Ref			
8–14	0.66	1.94	1.52 2.47	< 0.0001
≥ 15	1.36	3.91	2.65 5.75	< 0.0001
Missing	1.24	3.45	2.92 4.08	< 0.0001
**EuroQol-5D-5L**				
[− 0.1 to 0.6]	1.73	5.65	4.16 7.68	< 0.0001
(0.6–0.8]	0.94	2.57	1.91 3.46	< 0.0001
(0.8–1]	Ref			
Missing	1.85	6.35	4.85 8.30	< 0.0001
**EORTC-QLQ-C30**				
[0–54)	1.86	6.40	4.59 8.91	< 0.0001
(54–78]	1.20	3.34	2.54 4.39	< 0.0001
(78–88]	0.55	1.73	1.26 2.36	0.001
(88–100]	Ref			
Missing	1.60	4.97	3.95 6.27	< 0.0001

*HR* hazard ratio, *CI* confidence interval, *%* percentage, *HADS-Anxiety*, Hospital Anxiety and Depression questionnaire’s Anxiety subscale; *HADS-Depression*, Hospital Anxiety and Depression questionnaire’s Depression subscale; *EORTC-QLQ-C30*, The European Organisation for Research and Treatment of Cancer Quality of Life Questionnaire Core 30-Global Quality of Life scale

^a^pTNM: stage classified by the American Joint Committee on Cancer 7th edition TMN system: I, the cancer has grown through the mucosa and has invaded the muscular layer of the colon. No regional lymph node metastasis or distant metastasis exists; II, the cancer has grown through the wall of the colon, or through the layers of the muscle to the visceral peritoneum, or has grown into nearby structures. No regional lymph node metastasis or distant metastasis exists; III, metastasis in regional lymph nodes but no distant metastasis. Cases with metastasis in regional lymph nodes and distant metastasis (IV) were excluded from further analysis

^b^R-stage of the operation: residual tumor (R) classification: R0, complete absence of tumor; R1, microscopic evidence of residual tumor; R2, macroscopic evidence of the tumor/palliative care; these cases were excluded from further analysis

^c^ASA: anesthesia risk in relation to patient’s condition, class I–III, from healthy patients to those with a serious systemic but not disabling disease; and IV, patients with a disabling serious systemic disease

In the multivariable analysis, we identified predictors of mortality over the 5-year follow-up to be being older; being male; having a higher TNM stage; having a higher lymph node ratio; a result of CRC surgery classified as R1 or R2; invasion of neighboring organs; higher score on the Charlson comorbidity index; an ASA IV; and worse scores on the EORTC and EQ-5D health-related quality of life questionnaires, as compared to those with higher scores in each of those questionnaires (Table 4). Given the strong relationship between HADS and EORTC (chi-squared test *p*-value < 0.0001) and HADS and EQ-5D (chi-squared test *p*-value < 0.0001), HADs did not appear to be statistically significant in the multivariate model. The c-index of this model was 0.800 (0.796–0.806 95% bootstrap based confidence interval), for which the tenfold cross-validation provided a c-index of 0.801.

**Table 4:** Multivariate Cox proportional hazards regression analysis of predictors of mortality over a 5-year follow-up

**Variables**	**Estimate**	**HR**	**HR (95% CI)**	***p***	**Score**
**Charlson’s comorbidity index**					
1–2	Ref				0
3–4	0.15	1.16	0.98 1.38	0.078	0
≥ 5	0.78	2.18	1.74 2.74	< 0.0001	2
**Log odds of positive nodes ratio**					
[0–0.04)	Ref				0
[0.04–0.15)	0.24	1.27	0.94 1.72	0.124	0
[0.15–0.4)	0.65	1.92	1.42 2.58	< 0.0001	2
[0.4–1]	1.09	2.97	2.17 4.07	< 0.0001	3
Missing	0.54	1.72	1.26 2.34	0.001	2
**Gender**					
M	0.31	1.37	1.16 1.61	< 0.0001	1
F	Ref				0
**Age**					
< 70	Ref				0
[70, 80)	0.32	1.38	1.16 1.64	< 0.0001	1
≥ 80	0.90	2.46	2.02 3.00	< 0.0001	3
**pTNM**^**a**^					
0, I, II	Ref				0
III	0.27	1.31	0.99 1.75	0.063	0
IV	1.13	3.09	2.33 4.11	< 0.0001	4
**R stage**^**b**^					
R_0_	Ref				0
R_1_	0.45	1.56	1.21 2.03	0.001	1
R_2_	1.52	4.58	3.45 6.09	< 0.0001	5
Missing	0.31	1.37	0.94 2.00	0.104	0
**ASA**^**c**^					
I, II, III	Ref				0
IV	0.58	1.78	1.33 2.39	< 0.0001	2
Missing	0.59	1.80	1.27 2.54	0.001	2
**Invasion of other organs**					
0 or 1	Ref				0
2 or 3	0.44	1.56	1.28 1.90	< 0.0001	1
**EUROQOL-5D-5L**					
[− 0.1 to 0.6]	0.99	2.68	1.78 4.04	< 0.0001	3
(0.6–0.8]	0.65	1.91	1.36 2.67	< 0.0001	2
(0.8–1]	Ref				0
Missing	1.31	2.69	2.42 5.64	< 0.0001	4
**EORTC-QLQ-C30**					
[0–54)	0.88	2.42	1.56 3.75	< 0.0001	3
(54–78]	0.49	1.63	1.17 2.28	0.004	2
(78–88]	0.08	1.09	0.77 1.53	0.633	0
(88–100]	Ref				0
Missing	0.43	1.54	1.04 2.27	0.030	1

	**C-index**	**2.5% bootstrap quantile**	**97.5% bootstrap quantile**		
Proposed model	0.800	0.796	0.806		
Continuous score	0.794	0.789	0.799		
Categorized score	0.779	0.774	0.785		

*HR* hazard ratio, *CI* confidence interval, *%* percentage, *EORTC-QLQ-C30*, The European Organisation for Research and Treatment of Cancer Quality of Life Questionnaire Core 30-Global Quality of Life scale

^a^pTNM: stage classified by the American Joint Committee on Cancer 7th edition TMN system: I, the cancer has grown through the mucosa and has invaded the muscular layer of the colon. No regional lymph node metastasis or distant metastasis exists; II, the cancer has grown through the wall of the colon, or through the layers of the muscle to the visceral peritoneum, or has grown into nearby structures. No regional lymph node metastasis or distant metastasis exists; III, metastasis in regional lymph nodes but no distant metastasis. Cases with metastasis in regional lymph nodes and distant metastasis (IV) were excluded from further analysis

^b^R-stage of the operation: residual tumor (R) classification: R0, complete absence of tumor; R1, microscopic evidence of residual tumor; R2, macroscopic evidence of the tumor/palliative care; these cases were excluded from further analysis

^c^ASA: anesthesia risk in relation to patient’s condition, class I–III, from healthy patients to those with a serious systemic but not disabling disease; and IV, patients with a disabling serious systemic disease

**Fig. 1:**
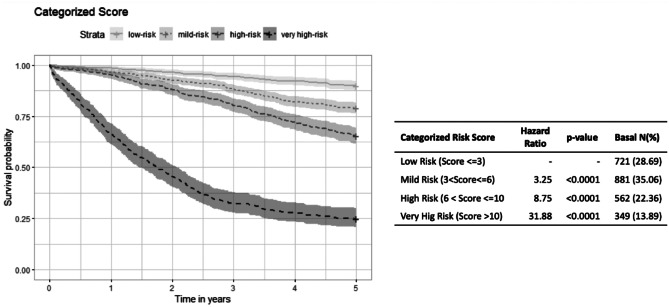
The Kaplan–Meier curves for the categorized risk score

Based on the variables identified in this multivariable model, and the weights assigned to each of the categories of their variables, a time-dependent mortality risk score was obtained for the 5 years of follow-up, which was categorized into four categories from lower to higher risk of death with 3, 6, and 10 as the cut-off points. A c-index of 0.794 and 0.779 was obtained for the continuous and categorized scores, respectively. The bootstrap validation provided bootstrap 95% confidence intervals for the c-index of (0.789–0.799) and (0.774–0.785) for the continuous and categorical scores, respectively (see Table 4). Figure 1 shows the Kaplan–Meier curves for the categorized score together with the hazard ratio for each category, where statistically significant differences between all risk categories were obtained.

## Discussion

In the current study, we demonstrated that HRQoL measurement was significantly associated with prognosis in CRC patients in a long-term follow-up. We likewise developed and validated a prognostic index for which higher index values were associated with a significantly higher risk of mortality at a given time during a 5-year follow-up. This score can be used in the clinical setting to stratify patients with CRC who underwent surgery into very high-, high-, intermediate-, and low-risk mortality groups in a 5-year follow-up time, combining clinical variables with HRQoL time-dependent measures. Our index showed good discrimination ability and was validated by bootstrap validation. It had a c-index of 0.794 and 0.779, for the continuous and categorized scores, respectively, indicating that it is a reliable tool for estimating prognosis in CRC patients. In addition, the proportional hazards assumption holds for both the model and the score.

Several international societies currently recommend the use of all-cause mortality risk prediction tools when making decisions regarding screening and treatment in geriatric oncology [35]. This recommendation extends to patients with CRC [3], which, despite an improved diagnosis due to greater availability of diagnostic techniques and advances in intraoperative postoperative care and medical care, still ranks as the second most common cancer cause of death worldwide. Therefore, identifying postoperative factors that help predict the prognosis in CRC patients is important—ideally, these would be factors that can be measured quickly, easily, and non-invasively in an ordinary clinical setting [3].

The sociodemographic and clinical characteristics of patients with CRC have been studied more frequently in relation to short- and medium-term mortality. In our study, among the patients’ sociodemographic characteristics, being older [2, 4, 36] and male [36] were related to higher mortality. Within the patients’ clinical parameter at baseline or after surgery, those related to the severity of the CRC, such as having a higher TNM stage [2], a higher lymph node ratio [37, 38], a result of CRC surgery classified as R1 or R2 [39], invasion of neighboring organs [17], or those related to comorbidities or life expectancy, such as a higher score on the Charlson four index [4, 40], or an ASA IV [5, 40–42], have been shown to be significant prognostic indicators in CRC patients.

HRQoL has been considered a potential predictor of survival for the general population [12] and also for cancer patients [43]. As recent publication state, [9, 44] HRQoL is a concept that has gained increasing importance as prognostic tool, having done more research in recent years regarding this topics. However, these are not sufficient, as most of them have just a short-medium-term follow-up [13, 14, 16, 44, 45], or taking into account only advanced stage of disease [13–16]. To our knowledge, only two studies have so far examined the association between HRQoL and mortality in long-term CRC survivors, with providing initial evidence for an inverse relation between better HRQoL and mortality [12, 17]. In those studies, patients were only contacted once after baseline, with HRQoL not being considered a time-dependent covariate in the survival analysis. However, HRQoL may vary over time. At the same time, they used a short and not widely used Veterans RAND 12-item Health Survey to assess HRQoL [12] or did not have information on comorbidities or on tumor stage [17].

In our study, we have collected information on patients’ perceived HRQoL during follow-up at different points in time, using a variety of tools, including a questionnaire, the EQ-5D, that captures general HRQoL, the EORTC, a more specific HRQoL tool for cancer patients, and a questionnaire, the HADS, which focuses on measuring levels of anxiety and depression. We found that having worse scores on the EORTC, and EQ-5D HRQoL questionnaires—i.e., worse quality of life measured by each of these questionnaires, compared to patients with better scores in each of those questionnaires—was related to a greater likelihood of dying in all periods of the follow-up. Our results therefore indicate that poorer scores on the EORTC, and EQ-5D, are also useful predictors of the CRC prognosis when used in addition to the previously described clinical and sociodemographic parameters. This is consistent with the previous studies mentioned above [12, 17].

Regarding the underlying mechanisms of the association between HRQoL and survival in cancer patients, we expanded Ratjen’s analysis [17], adjusting our analyses for tumor stage, recurrence, and comorbidities—variables that might influence worse HRQoL. Our results thus showed that HRQoL is an independent predictor of a prognosis of survival. Moreover, in our index, a low HRQoL score ranked higher (3 points) than variables such as having 5 or more comorbidities (2 points) or invasion (1 point).

Regarding HADS, in our sample, in the univariable analysis, greater anxiety and depression were associated with a greater risk of dying, but they do not appear as independent predictors in the multivariate analysis. The strong association we found in our cohort between HADS and the EORTC and EQ-5D HRQoL scores may be an explanation. An alternative explanation could be that those with worse HADS scores have more severe CRC or more comorbid conditions, which at the same time affect the patients’ survival. Consistent with this observation, Adams et al. [12], who evaluated physical and mental component scores with the Veterans RAND 12-item Health Survey, observed a stronger association between mortality and physical component than with mental component. This contrasts with the potential explications of Ratjen et al. [17] for the observed association between HRQOL and survival; they suggested that it might be psychological distress (stress and depression) that influenced HRQoL. A key factor behind effective coping with cancer-related problems is resilience and it has been considered a protective factor against different health problems, including colon cancer. The higher the resilience, the lower the vulnerability and risk of illness and the better the quality of life [46, 47]. A feasible option for future studies would be to investigate in greater depth the relationship between resilience, HRQoL, and mortality in CRC patients.

To date, no prognostic index has been created considering PROMs variables. Lee et al. [48] stated that function status is particularly useful in prognostic systems because it reflects the severity and consequences of disease, since it is a marker at different points throughout the patients’ trajectories. We believe that this could be extended to HRQoL measurements. The prognostic model of Tanio et al. [3], which combines lymphocyte, monocyte, and neutrophil with clinical characteristics, has an AUC of 0.642. The inclusion of HRQoL measures might perhaps be the reason that our discrimination index compares favorably with Tanio’s model.

Strengths of our study include a large cohort with a variety of clinical information collected through a 5-year follow-up, including patients’ perception of their HRQoL. In developing the model, we followed the TRIPOP guidelines [49], using robust statistical techniques that took into account the various assessments of the HRQoL of patients throughout the 5-year follow-up period. Finally, another strength was the use of valid, reliable, and widely used HRQoL questionnaires. The EORTC-QLQ-C30 is one of the most widely used cancer-specific instrument and EQ-5D-5L is one of the most widely used general instruments.

Several limitations of this study must also be acknowledged. First, as with all prospective cohort studies, loss of patients and information at follow-up is inevitable. Although we have tried to reduce it to a minimum, with such a long follow-up, some loss of data is inevitable. Secondly, all predictive indices tend to optimize the fit of the data to a captive population and, although our score fitted the data well in both the development and validation sets, it is important to cross-validate the scoring system externally by applying the model to a different population in order to assess its predictive power [7]. Finally, some other possible predictors such as molecular data, or the WHO performance status, were not included in the study.

In summary, we identified certain pre-surgery clinical and sociodemographic factors in CCR patients which, combined with the patients’ HRQoL perception in a long-term follow-up, can be used in a prognostic index that has several potential uses in clinical and research fields. It is an accurate tool, indicating that it could be routinely used for better treatment. It may be useful in follow-up, distinguishing both high- and low-risk patients and thus identifying patients with whom it is especially important to discuss advance directives and to provide help in the treatment strategy and in the prevention of adverse outcomes.

### Supplementary Information

Below is the link to the electronic supplementary material.

Supplementary file1 (PDF 123 KB)

## Data Availability

Data available upon request.
