# Correction to: Five-year follow-up mortality prognostic index for colorectal patients

**DOI:** 10.1007/s00384-023-04472-z

**Published:** 2023-06-24

**Authors:** Miren Orive, Irantzu Barrio, Santiago Lázaro, Nerea Gonzalez, Marisa Bare, Nerea Fernandez de Larrea, Maximino Redondo, Sarai Cortajarena, Amaia Bilbao, Urko Aguirre, Cristina Sarasqueta, José M. Quintana, for the REDISSEC-CARESS/CCR group

**Affiliations:** 1 Department of Social Psychology, Faculty of Pharmacy, University of the Basque Country UPV/EHU, Vitoria, Spain; 2 Department of Mathematics, University of the Basque Country UPV/EHU, Leioa, Spain; 3 Servicio de Cirugía General, Hospital de Basurto, Bilbao, Spain; 4 Unidad de Investigación, Hospital de Galdakao-Usansolo, Galdakao, Spain; 5 Unidad de Epidemiología Clínica, Corporacio Parc Tauli, Barcelona, Spain; 6 Centro Nacional de Epidemiología ISCIII, Madrid, Spain; 7 Unidad de Investigación, Hospital Costa del Sol, Malaga, Spain; 8 Unidad de Investigación, Hospital de Basurto, Bilbao, Spain; 9 Unidad de Investigación, Hospital Donostia/BioDonostia, Donostia, Spain; 10 Red de Investigación en Servicios Sanitarios y Enfermedades Crónicas (REDISSEC), Galdakao, Spain; 11 CIBER Epidemiología y Salud Pública (CIBERESP), Madrid, Spain; 12 Red de Investigación en Cronicidad, Atención Primaria y Promoción de la Salud (RICAPPS), Galdakao, Spain; 13 Basque Center for Applied Mathematics, BCAM, Bilbao, Spain; 14 Kronikgune Institute for Health Services Research, Barakaldo, Spain

**Correction to: International Journal of Colorectal Disease (2023) 38:64** **https://doi.org/10.1007/s00384-023-04358-0**

The Funding information in the published version of the above article was incomplete. The complete information is presented as follows:

**Funding** Open Access funding provided thanks to the CRUE-CSIC agreement with Springer Nature. This work was supported in part by grants from the Instituto de Salud Carlos III and the European Regional Development Fund (PS09/00314, PS09/00910, PS09/00746, PS09/00805, PI09/90460, PI09/90490, PI09/90453, PI09/90441, PI09/90397); the Spanish Ministry of the Economy (PID2020-115738 GB-I00); the Departments of Health (2010111098) and Education, Language Policy and Culture (IT1456-22; IT1598-22; IT-1187–19) of the Basque Government; the Research Committee of Galdakao Hospital; the REDISSEC (Red de Investigación en Servicios de Salud en Enfermedades Crónicas) thematic network of the Instituto de Salud Carlos III; and the Department of Education of the Basque Government through the Consolidated Research Group MATHMODE (IT1456-22) and the Basque Government through BMTF “Mathematical Modeling Applied to Health” Project. This study has been funded by Instituto de Salud Carlos III (ISCIII) through the projects "RD16/0001/0001" and RD21CIII/0003/0017 and co-funded by the European Union

The original article has been corrected.

