# Supplementary material for: Five-year follow-up mortality prognostic index for colorectal patients
Source: Int J Colorectal Dis. 2023 Mar 9;38(1):64. doi: 10.1007/s00384-023-04358-0 (PMC9998584; doi:10.1007/s00384-023-04358-0)

Supplementary online Figure 1. Flowchart describing the cohort evolution during the five years of follow-up

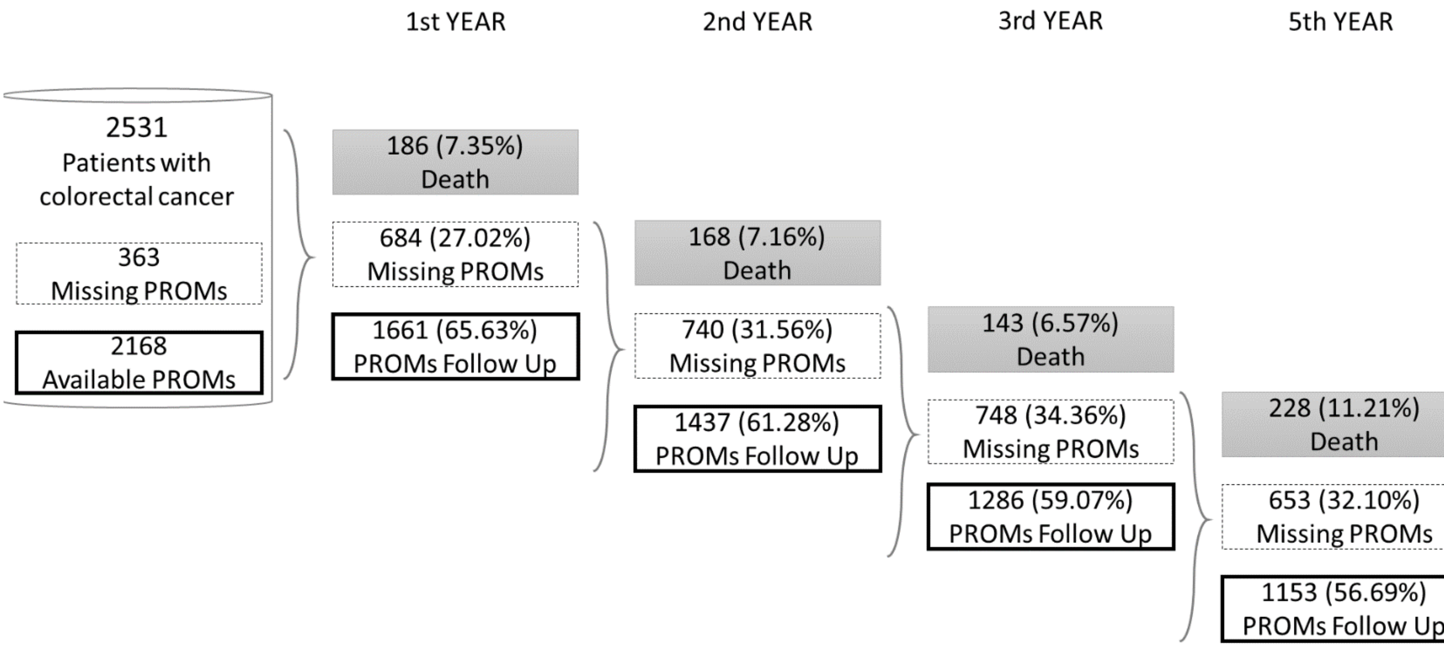

Supplement: Supplementary file 1 — Supplementary file1 (PDF 123 KB) [file 384_2023_4358_MOESM1_ESM.pdf]
